# Comparison of the Nutritional Status of Swiss Albino Mice Fed on Either a Purified or Cereal-Based Diet for 15 weeks

**DOI:** 10.1155/2023/9121174

**Published:** 2023-05-31

**Authors:** Hellen W. Kinyi, Charles Drago Kato, Deusdedit Tusubira, Gertrude N. Kiwanuka

**Affiliations:** ^1^Department of Biochemistry, Faculty of Medicine, Mbarara University of Science and Technology, P.O. Box 1410, Mbarara, Uganda; ^2^Department of Biochemistry, School of Medicine, Kabale University, P.O. Box 317, Kabale, Uganda; ^3^School of Biosecurity, Biotechnical and Laboratory Studies, College of Veterinary Medicine, Animal Resource and Biosecurity, Makerere University, P.O. Box 7062, Kampala, Uganda

## Abstract

**Background:**

Laboratory animals are commonly fed on cereal-based diets (CBDs) whose nutrient composition is unknown and may confound the metabolic response to study interventions. Purified diets such as AIN-93M are therefore recommended, as their nutrient composition is known. However, few studies have evaluated their use as adequate control diets. The aim of this study was to compare the nutrition status of Swiss albino mice fed on either CBD or AIN-93M for 15 weeks.

**Methods:**

Twenty Swiss albino mice aged 6–8 weeks and weighing 21.7 g ± 0.6 were fed on either CBD or AIN-93M diet for 15 weeks. Their nutritional status was evaluated using anthropometric and hematological indices, serum glucose, total protein, albumin, and total cholesterol to select an appropriate normal control diet.

**Results:**

The CBD had low-calorie content (2.57 kcal/g) and protein (11 ± 3.8 g/100 g) compared to AIN-93M (3.8 kcal/g and 14 g/100 g, respectively). The BMI of male mice fed on CBD and AIN-93M diets was significantly higher (*P*=0.0139 and *P*=0.0325, respectively) compared to that of females fed on similar diets. Animals in the CBD group had lower hemoglobin (15.1–16.9 g/dl) compared to those in the AIN-93M group (18.1–20.8 g/dl). Serum albumin levels were higher in both male (*P*=0.001) and female (*P*=3 × 10^−6^) mice fed on AIN-93M compared to those fed on CBD. Females in the AIN-93M group had higher cholesterol (*P*=0.026) than those in the CBD group.

**Conclusion:**

The AIN-93 diet of caloric value 3.85 kcal/g (total protein 14 g, total fat 4 g of soy bean oil, fibre 5 g, and total carbohydrate 42 g per 100 g) can be safely used as a normal control diet in long-term research studies using Swiss albino mice.

## 1. Introduction

The diet of laboratory animals is a dominant environmental factor which interacts with genes to influence their physiology, metabolism, and behaviour both in health and disease [1, 2]. These diets contain a combination of macro- and micronutrients necessary for development, reproduction, and metabolism [3]. The nutrient composition of diets can therefore alter metabolism causing changes in levels of blood markers that may indicate risk of developing certain diseases [1]. It is therefore important to properly control laboratory diets to be able to draw correct conclusions of the effect of a particular biomedical intervention [4].

Laboratory rodent diets are classified into chow, purified, or chemically defined diets [5]. Chows are also known as grain or cereal-based, unrefined, or nonpurified diets [4, 6]. They have been in use since the 1940s as an inexpensive control or maintenance diet, which is generally considered sufficient to maintain the animals' health [2], though there is usually no control used to substantiate this claim. Chows are able to provide adequate nutrition to lab animals because they contain multiple ingredients such as corn, oats, soybean meal, wheat, cotton seeds, shellfish, maize bran, salt, sunflower seeds, bone meal, vitamin, and mineral premixes each of which provide many nutrients [1, 4, 7]. However, differences in the extent of processing of ingredients, differing conditions, and locations of where they are harvested and stored are thought to contribute to the variation in the results generated from animals fed on chows [8]. Additionally, most chows are marketed as closed formula commercial diets, that is, the formula is kept a secret by the manufacturer, and their nutrient content may vary from batch to batch and may contain non-nutritive components such as phytoestrogen and environmental contaminants such as arsenic [1, 9, 10].

On the other hand, purified animal diets are composed of refined ingredients which serve as a source of a single nutrient, for example, isolated proteins such as casein, corn starch, sucrose, maltodextrin, soybean oil, purified vitamins, and minerals [11, 12]. Purified diets are well defined and have open formulas that have minimal variability from batch to batch [2, 9]. They have been shown to improve reproducibility of animal studies [13]. Since they allow modification of individual nutrients, they can be used to create pathological status such as deficiencies or excesses as well as study nutrient-toxin relationships [2, 12]. Nevertheless, they have the disadvantages of being low in soluble fibre, reduced palatability, and increased costs of production [9, 13, 14]. The National Institute of Health (NIH) and the American Institute of Nutrition (AIN) have developed several open source purified diet formulas [9, 15].

Although preclinical animal studies help us gain insight into disease mechanisms as well as guide our selection of management plans for clinical trials, they are marred with poor reproducibility [2, 16]. Varied nutrition background, due to cereal-based diets resulting in unknown nutrient interactions is thought to contribute to the poor reproducibility commonly seen in nutrition and metabolism studies [2]. There is a wealth of information showing that purified diets such as AIN-93M reduce variability in results between laboratories, can be used as reliable controls in nutrition studies, and may yield animals with better nutrition status [17]. However, there are few studies that validate the use of purified diets as adequate control diet in animal studies. Therefore, this study validated the use of AIN-93M as a control diet for Swiss albino mice fed on a cereal-based or purified AIN-93M diet for 15 weeks.

## 2. Materials and Methods

### 2.1. Experimental Design

For the study, Swiss albino mice aged 6–8 weeks and with a mean weight of 21.7 g ± 0.6 were used. Ten mice of same sex were housed in metabolic cages measuring 35 × 30 × 15 cm and maintained at room temperature of 20–25°C and relative room humidity of 70–80%. The beddings were an inch of wood shavings, changed weekly to reduce odor and excessive growth of pathogens. All procedures used were in accordance with guidelines from the care and use of laboratory animals [18] following the Mbarara University institutional ethics approval (study no. 19/08-20). The study was registered by the Uganda National Council of Science and Technology (NS159ES).

The experimental animals were divided into 2 groups, namely, CBD and AIN-93M. Each group had 20 animals, 10 males, and 10 females. The animals were fed the respective diets for a period of 15 weeks. Mice were provided with fresh water and food daily, husks were changed every four days, and biweekly body weight measurements were recorded. Manual weighing of left-over food after 24 hours was used to estimate how much food was consumed by animals from each cage. Initially, each cage containing 10 animals of either sex was provided 50 g of food/24 hours. However, it was noted that there was no left-over food in cages fed on CBD. The food provided was progressively increased to 80 g/day. This did not account for food spilled, crumbled, defecated, or urinated on. At the end of the experiment, at total of 7.7 kg of CBD and 4.8 kg of AIN-93M had been provided to the animals.

### 2.2. Weight Assessment and Determination of BMI

The weight of the animals in each group was taken biweekly using an electronic balance (Kern PCB, Germany), and the value recorded to the nearest 0.1 g. The length of the animals was determined by measuring the nasal to anal distance to the nearest 0.1 mm using a caliper and ruler. To facilitate this, the animals had been anesthetized as described below [19]. The body mass index was calculated as the ratio between body weight and square surface area (g/m^2^) [19].

The body surface area was derived from the DuBois equation [19] as follows:

(1) $$\begin{array}{c} body\;surface\left(m^{2}\right)=0.007184\times weight\;{\left(kg\right)}^{0.425}\times height\;{\left(cm\right)}^{0.725}\text{.} \end{array}$$

### 2.3. Sample Collection and Analysis

The animals were anesthetized using intraperitoneal injection of a mixture of 16 mg xylazine and 60 mg ketamine and 1.0 ml of blood collected from the inferior vena cava (IVC) as described previously [20]. Blood was collected into either an ethylenediaminetetraacetic acid (EDTA) vacutainer for hematology analysis or a plain vacutainer for biochemical analysis. Blood collected in plain vacutainers was stored at room temperature for 1 hour, followed by centrifugation at 3000 rpm (1500 ×g) for 15 min to obtain serum.

Complete blood count (CBC) was determined using an automated hematological analyzer, Coulter CBC-5 Hematology Analyzer (Beckman Coulter, China). Serum glucose was determined using on-call plus blood glucose test strips and glucometer (ACON Laboratories USA). Total protein, serum albumin, and cholesterol were determined using Beckmann Coulter AU 480 Chemistry Analyzer following manufacturers' instructions supplied with the kits (Vitro Scient, Hannover Germany).

### 2.4. Experimental Diets

For the CBD, a 50 kg sack (Batch Number 93486) of Nuvita mice pellets manufactured by Engaano Millers Limited, Jinja Uganda East Africa was procured. AIN-93M diet was formulated using food grade ingredients in the formula of America Institute of Nutrition [9]. Vitamin-free casein was obtained from Amos Dairies, Uganda Limited and soya bean oil from Arma Oils, Egypt. The carbohydrates were supplied by food grade cornstarch from Nakash Chemicals International Gujarat, India, and maltodextrin from Bluecraft Agro Gujarat, India. Sucrose and carboxymethylcellulose (CMC) were purchased from Loba Chemie PVT Limited, Mumbai India. AIN-93M vitamin and mineral mixes were supplied by MP Biomedical LLC Fountain Parkway USA.

Nutrition composition of CBD pellets was analyzed according to the protocols of the Association of Official Analytical Chemists (AOAC) for crude fibre, crude fat, and crude protein content [21]. The carbohydrate was calculated by subtracting the sum (g/100 g dry matter) of crude protein, crude fat, ash, and fibre from 100 g [22]. The caloric value was the sum of the percentages of proteins and carbohydrates multiplied by a factor of 4 (kcal/g) and total lipids multiplied by a factor of 9 (kcal/g) [22].

### 2.5. Data Management and Analysis

Data collected for each experiment were entered in excel spread sheet and later transferred to Graph pad 6.0 statistical software for further analysis. The data for each group were expressed as mean ± SD and subjected to normality testing using Shapiro Wilk test. Comparison across the 2 male and 2 female groups fed on either CBD or AIN-93M diet was analyzed using two-way ANOVA without replication followed by Tukey or Dunnets multiple comparisons test set at a significance level *P* < 0.05.

## 3. Results

### 3.1. Proximate Composition of the Diets

Cereal-based diet had low-calorie content (2.57 kcal/g) compared to the AIN-93M diet (3.8 kcal/g). The main source of calories for both diets was carbohydrates. The dry weight amounts of the two diets are presented in Table 1.

### 3.2. Biweekly Body Weight and BMI

**Figure 1:**
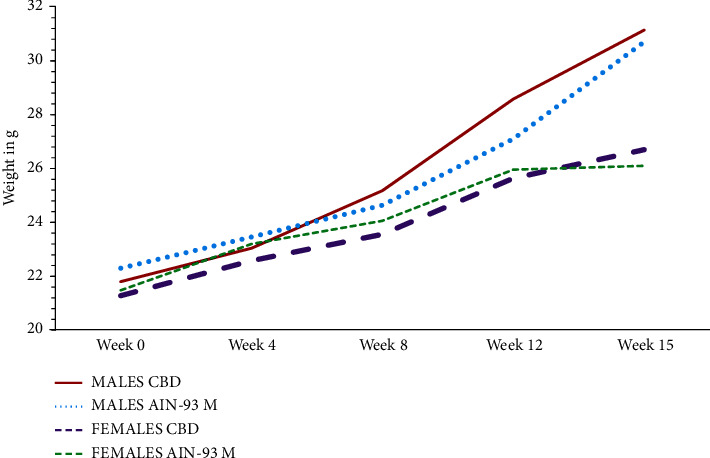
Changes in the body weight of Swiss albino mice fed on CBD and on AIN-93M diet for 15 weeks. The effect of the type of diet on body weight is evident from week 10.

**Figure 2:**
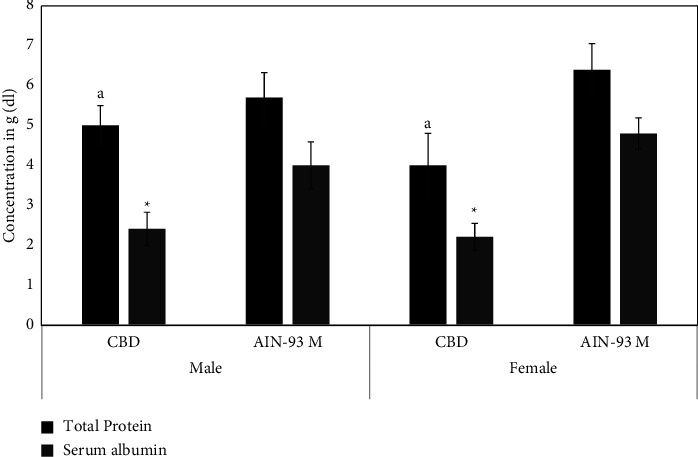
Total serum protein and albumin of Swiss albino mice fed on AIN-93M and CBD for 15 weeks. Data are expressed as mean ± SD (*n* = 5). ^*∗*^indicates statistically significant differences from AIN-93M of same gender (*P* < 0.05) while *a* indicates statistically significant differences from AIN-93M females (*P* < 0.05).

On average, male mice had an overall weight gain of 6.7 ± 2.4 g over the duration of the experiment while females gained 5 ± 1.0 g as shown in Figure 1. There was an increase in the biweekly average weight as shown in (Figure 2). Significant weight increase in males was recorded from week 8 to 14 for those fed on CBD and from week 10 to 14 for those fed on AIN-93M. However, significant weight changes were noted from week 12 to 14 for females in both the AIN-93M and CBD groups (*P*-values in Supplementary file 1). There was a significant difference in body weight of male and female animals fed on both diets (*F*_2,8_ = 19.7, *P*=0.0008) on week 15. Males fed on CBD were not significantly heavier than those fed on AIN-93M (CBD 31.14 ± 1.9 g, AIN-93M 30.74 ± 1.4 g; *P*=0.97). Similarly, there was no significant difference in the weights of females fed on either diet on week 15 (CBD 26.7 ± 1.5, AIN-93M 26.1 ± 0.8, *P*=0.9124). However, males fed on CBD and AIN-93M were heavier (*P*=0.001 and *P*=0.0006, respectively) than females fed on similar diets.

There was a statistically significant difference (*F*_3,12_ = 15.7, *P*=0.00019) in the BMI of male mice (CBD 3.7 ± 0.1 g/cm^2^; AIN-93M 3.4 ± 0.9 g/cm^2^) and female mice (CBD 3.5 ± 0.9 g/cm^2^; AIN-93M 3.3 ± 0.9 g/cm^2^). BMI of female mice fed on CBD was lower (*P*=0.0139) than that of male mice fed on CBD. Similarly, female mice fed on AIN-93M had lower BMI (*P*=0.03) than male mice fed on the same diet. However, there was no statistically significant difference in the BMI of animals of similar sex fed on different diets (male *P*=0.109 and female *P*=0.12).

### 3.3. Hematological Indices

Female mice in the AIN-93M group had a higher RBC count compared to those in the male CBD group (*P*=0.013). Hemoglobin level of both male and female animals in the CBD group was lower than that of animals of similar gender in the AIN-93M group (*P*=2.6 × 10^−5^ and *P*=3.01 × 10^−5^, respectively). Similarly, the mean corpuscular hemoglobin (MCH) and mean corpuscular hemoglobin concentration (MCHC) were lower in the CBD group than those in the AIN-93M (Table 2).

Regardless of the type of diet, female mice had higher white blood cell (WBC) count than the males. However, only the female mice in AIN-93M group had significantly higher total white blood cell and lymphocyte count than animals in the other groups. The WBC count was within the normal count for mice (Table 3).

### 3.4. Random Blood Glucose and Total Serum Cholesterol

The random blood glucose level of mice fed on CBD (Male 6.34 mmol/L; Female 6.4 mmol/L) was not significantly different from those fed on AIN-93M (Male 6.2 mmol/L; Female 6.14 mmol/l) (*F*_3,12_ = 0.12; *P*=0.946).

Similarly, there was no statistically significant difference in the average total serum cholesterol of male mice fed on either CBD (97 ± 9.1 mg/dl) or AIN-93M (93 ± 8.2 mg/dl) and females fed on CBD (92 ± 7.8 mg/dl) and AIN-93M (104 ± 6.1 mg/dl) (*F*_3,12_ = 2.13; *P*=0.15).

### 3.5. Total Protein and Serum Albumin Levels

There was a significant difference in the total protein across the four groups (*F*_3,12_ = 12.2; *P*=0.0006). Total protein of female mice in the AIN-93M group was higher than that of both female (*P*=0.0002) and male (*P*=0.02) mice in the CBD group.

Similarly, there was a significant difference in the total serum albumin levels across the four dietary groups (*F*_3,12_ = 34.9; *P*=3.27 × 10^−6^). Male mice fed on CBD diet had lower serum albumin compared to those fed on AIN-93M (*P*=0.00012). Female mice fed on CBD also had lower serum albumin than those fed on AIN-93M (*P*=3.77 × 10^−7^).

## 4. Discussion

The rise of nutrition-related diseases such as diabetes mellitus, obesity, hypertension, and their comorbidities has created the need for animal research on treatment interventions and disease mechanisms. Mice are widely used as animal models in biomedical research, and manipulation of diet is the most effective way of regulating their health status, growth rate, reproduction, and survival rates [12, 15]. We found that animals fed on either CBD or AIN-93M had similar weight. The hemoglobin concentration, total protein, and serum albumin levels of animals fed on AIN-93M were higher compared to those fed on CBD. These variations could alter animal metabolism and influence their response to various biomedical interventions in research.

The National Research Council (US) recommends a mouse diet of 3.9 kcal/g mostly supplied by carbohydrates [8, 24]. Low-calorie diets cause increased compensatory feeding to meet both energy and nutrient requirements which is limited by gastrointestinal capacity and may result in under nutrition [7]. Although the CBD had low calories per Gram compared to the AIN-93M diet, the lack of differences in biweekly weights and BMI of animals maintained on the two different diets for 15 weeks suggests that mice regulate their food intake within limits dictated by the macronutrient and caloric content in the diet. This is supported by the increased feed intake by mice maintained on CBD implying that high amount is required to keep mice healthy. The downside to this is the increased financial burden to the laboratory.

Carbohydrates are preferred sources of energy in rodents as typified by starch-based diets consumed in the wild. Although the CBD had lower carbohydrate content than AIN-93M, there is no associated hypoglycaemia in animals fed on this diet as glucose can be formed from other metabolites through the process of gluconeogenesis [25]. Both diets had adequate lipid content. Diets containing 0.5–40% lipids have been shown to support adequate weight in gain in mice [7, 24]. Adult mice require 12–18% crude protein in diet to remain healthy [7, 24]; however, this requirement is higher in pregnancy and lactation. The low protein content of CBD coupled with other quality factors such as digestibility and amino acid score may be responsible for the low total protein and serum albumin in animals fed on this diet.

Animal weight is used to assess animal welfare and food intake as well as to compare effects of nutritional interventions [19, 26]. Generally, male mice weighed heavier than female mice, and this pattern is seen in this study and other similar studies [27]. The progressive weight gain seen in the study shows that the animals' welfare and food intake were adequate during the period of the study. This correlates with expected postmaturity weight gain of adult rodents [28]. The BMI values for females in this study were similar to those found in [19] for female C57BL/6J mice fed on normal diet aged over 20 weeks.

The evaluation of hematological and biochemical parameters is important in establishing the physiological status of an organism. They are used as biomarkers of nutritional status of an organism and in diagnosing infections and disease as well as organ injuries [27, 29, 30]. Female animals on both diets had higher RBC count, hemoglobin (Hb) concentration, and hematocrit than their male counterparts. Similar findings have been found elsewhere [27, 31]. The RBC count, hematocrit, and mean corpuscular volume found in this study match those of Santos et al. [30], in which animals were also fed on AIN-93M. However, these values are lower than those in which animals were fed on a commercial diet [27]. Variation in dietary composition could explain this discrepancy. Animals in both the CBD and AIN-93M groups in this study had higher Hb concentration than others in similar studies [27, 32]. Nonetheless, hematological values were within the normal ranges for mice as reported previously [33]. The low Hb and MCHC in animals in the CBD group were possibly because of low dietary protein. Low dietary protein reduces the synthesis of the globulin portion, resulting in low hemoglobin even in the presence of adequate iron.

Leucocytes participate in immunity and mediate both innate and adaptive responses. The total leucocyte counts of animals on both diets were within the normal range of 2 to 10 × 10^9^ cells/L(33). However, the values were lower than those reported in previous studies [27, 29, 32]. Variations could be due to the difference in the environment and diets. In mice, lymphocytes contribute 70–80% of the WBC count [33], and this was evident in this study. However, the neutrophils were slightly below the expected range of 20–30%. Neutrophils are made in the bone marrow and their number in blood therefore depends on the rate of synthesis and release from bone marrow. Other factors that influence the neutrophil count are their distribution between tissues and their rate of survival in blood and tissues [33, 34].

Biochemical parameters provide useful information about general nutritional and clinical status to identify specific nutritional deficiencies [35]. These markers have the advantage of detecting early changes in nutritional status before overt clinical features. Plasma proteins such as albumin participate in regulation of blood pH and transport and provide colloid osmotic pressure [36]. The total protein and serum albumin values in this study are higher for mice fed on AIN-93M than the values of 3.2–5.9 mg/dl and 2.5–3.4 mg/dl, respectively, from previous studies [27, 30]. Dietary protein intake determines the level of total protein in blood [36, 37]. Albumin contributes 30–50% of the total serum protein, and the quality of dietary proteins influences its serum level [37]. Cysteine supplementation is associated with increased serum albumin and could explain the higher serum albumin levels in mice fed on AIN-93M [38, 39].

The random blood sugar measures the amount of glucose circulating in blood, generally from diet [40]. The range of blood glucose values in this study (6.2–6.34 mmol/L) was lower than those from previous studies (7.2–12 mmol/L) [27, 32]. Blood glucose homeostasis is regulated by insulin synthesized by the pancreas, and disruptions in this regulation can be identified by RBS [40]. However, random blood sugar levels are influenced by the circadian clock as well as the degree of food intake [41]. This would explain the difference in blood sugar levels seen in this study compared to others.

Cholesterol is an important cellular compound with roles in cell membrane structure, steroid hormones, and bile acids synthesis [7]. Both dietary and de novo-synthesized contribute to the total serum cholesterol. Diets rich in saturated fatty acids are associated with increased serum cholesterol while those rich in unsaturated fatty acids such as soyabean oil have lower circulating cholesterol [42, 43]. The cholesterol values obtained in this study were within the range (80–150 mg/dl) of previous studies [27, 30]. The source of fat for AIN-93M diet is soyabean oil rich in linoleic and linolenic acid which have a hypocholesterolemia effect [43].

## 5. Conclusion

Many labs routinely use food intake and weight to assess the well-being of experimental animals. Results from this study have shown that although weight, blood sugar, and cholesterol of animals fed on CBD is similar to those fed the AIN-93M diet, the hemoglobin levels, total protein, and serum albumin were lower. Our data show that cereal-based diets may not provide enough proteins needed for efficient production of erythrocytes and albumin, which can potentially affect many physiological processes. On the other hand, AIN-93M was found to have the advantage of keeping mice with a normal body weight and adequate hematological and biochemical indices which is good for the general wellbeing of the animals. In view of these results, we confirm that AIN-93 is an appropriate normal control diet that should be used in animal studies, even those conducted in Africa. We recommend further studies to explore the cost implications of the use of the purified diets.

## Figures and Tables

**Table 1:** Nutrient composition of CBD and AIN-93M as dry weight basis.

Nutrient	CBD	AIN-93M
Protein	11 ± 3.8	14
Fat	5 ± 0.8	4
Fibre	15 ± 4.9	5
Total carbohydrates	42 ± 4.3	42
Gross energy (kcal/g)	2.57	3.85

DW is dry weight of nutrients in g/100 g of the diet.

**Table 2:** Hematological indices of mice fed CBD or AIN-93M diet for 15 weeks.

Diet	CBD		AIN-93M		
Gender	Male (*n* = 5)	Female (*n* = 5)	Male (*n* = 5)	Female (*n* = 5)	*P* values
Mean RBC count ∗ 10^12^/L	8.15 ± 0.2	8.64 ± 0.4	8.43 ± 0.8	9.09 ± 0.2^b^	(*F*_3,12_ = 4.4; *P*=0.026)
Mean hemoglobin (g/dl)	15.4 ± 0.3^*∗*^	15.82 ± 1.1^*∗*^	19.1 ± 1.0	19.4 ± 1.2	(*F*_3,12_ = 31.8; *P*=5.4 × 10^−6^)
Mean hematocrit (%)	36.1 ± 2.2	36.3 ± 3.0	38.0 ± 3.5	40.21 ± 2.4	(*F*_3,12_ = 2.3; *P*=0.128)
Mean corpuscular volume (fL)	42 ± 1.5^*∗*^^a^	44.8 ± 0.7	44.9 ± 0.8	44.2 ± 2.0	(*F*_3,12_ = 5.1; *P*=0.017)
Mean corpuscular hemoglobin (pg)	19.68 ± 0.7^*∗*^	19.3 ± 1.0^*∗*^	22.8 ± 0.9	21.4 ± 0.9	(*F*_3,12_ = 16.9; *P*=0.00013)
Mean corpuscular hemoglobin concentration (%)	42.9 ± 2.5^*∗*^	43.4 ± 0.7^*∗*^	50.4 ± 2.3	48.3 ± 0.8	(*F*_3,12_ = 22.6; *P*=3.2 × 10^−5^)

^*∗*^Values differ significantly from AIN-93M (*P* < 0.05); ^a^values differ significantly from CBD females (*P* < 0.05). ^b^values differ significantly from CBD males (*P* < 0.05).

**Table 3:** White blood indices of mice fed on CBD or AIN-93M diet for 15 weeks.

Parameter	CBD		AIN-93M		
Gender (*n* = 5)	Male	Female	Male	Female	*P* values
White blood cell count ∗ 10^9^/L	2.05 ± 0.2^a^	2.55 ± 0.5^a^	2.38 ± 0.4^a^	3.15 ± 0.4	(*F*_3,12_ = 5.9; *P*=0.01)
Lymphocyte count ∗ 10^9^/L	1.53 ± 0.2^a^	1.79 ± 0.2^a^	1.82 ± 0.4^a^	2.51 ± 0.4	(*F*_3,12_ = 10.6; *P*=0.001)
Neutrophil count ∗ 10^9^/L	0.39 ± 0.12	0.47 ± 0.09	0.43 ± 0.15	0.48 ± 0.09	(*F*_3,12_ = 0.9; *P*=0.463)
Mixed count ∗ 10^9^/L (monocytes, basophils, eosinophils)	0.13 ± 0.05	0.15 ± 0.04	0.13 ± 0.03	0.16 ± 0.02	(*F*_3,12_ = 0.87; *P*=0.478)

^a^Values differ significantly from AIN-93M females (*P* < 0.05); typical WBC count in mice is 2–10 × 10^9^ per litre [23].

## Data Availability

The datasets used and/or analyzed during the current study are included within the manuscript.

## Abbreviations

CBD: Cereal-based diet
AIN-93M: American Institute of Nutrition-93 Maintenance diet
BMI: Body mass index
CBC: Complete blood count.

## Supplementary Materials

Supplementary Materials Supplementary file 1 contains mean biweekly weights of male and female mice fed on either CBD or AIN-93M diets for 15 weeks. These data are used to plot Figure 1. The file also contains *P* values of Tukey's pairwise comparison of biweekly mean weight to their mean weight on day 1.
