# Supplementary material for: Comparison of the Nutritional Status of Swiss Albino Mice Fed on Either a Purified or Cereal-Based Diet for 15 weeks
Source: Biochem Res Int. 2023 May 31;2023:9121174. doi: 10.1155/2023/9121174 (PMC10247330; doi:10.1155/2023/9121174)
Supplement: Supplementary Materials — Supplementary file 1 contains mean biweekly weights of male and female mice fed on either CBD or AIN-93M diets for 15 weeks. These data are used to plot Figure 1. The file also contains P values of Tukey's pairwise comparison of biweekly mean weight to their mean weight on day 1. [file 9121174.f1.docx]

**Supplemental Information**

**Comparison of the nutritional status of Swiss albino mice fed on either a purified or cereal-based diet for 15 weeks**

Authors: Hellen W. Kinyi, Charles Drago Kato, Deusdedit Tusubira and Gertrude N. Kiwanuka

**INDEX TO SUPPLEMENTAL INFORMATION**

**Supplemental Tables**

**Table S1:** Mean biweekly weights (g) of Male mice fed on either CBD or AIN-93M for 15 weeks

**Table S1a:** P-values of Tukey's pairwise comparison of biweekly weight of male mice to the weight on day 1

**Table S2:** Mean biweekly weights (g) of Female mice fed on either CBD or AIN-93M for 15 weeks

**Table S2a:** P-values of Tukey's pairwise comparison of biweekly weight of female mice to the weight on day 1

**Table S1 related to Figure 1:** Mean biweekly weights (g) of Male mice fed on either CBD or AIN-93M for 15 weeks

|  | **Day 1** | | **Week 2** | | **Week 4** | | **Week 6** | | **Week 8** | | **Week 10** | | **Week 12** | | **Week 14** | |
| --- | --- | --- | --- | --- | --- | --- | --- | --- | --- | --- | --- | --- | --- | --- | --- | --- |
| **Animals** | **CBD** | **AIN-93M** | **CBD** | **AIN-93M 93M** | **CBD** | **AIN-93M 93M** | **CBD** | **AIN-93M 93M** | **CBD** | **AIN-93M 93M** | **CBD** | **AIN-93M 93M** | **CBD** | **AIN-93M 93M** | **CBD** | **AIN-93M 93M** |
| **Average** | **21.8** | **22.3** | **22.48** | **21.14** | **23.04** | **23.46** | **25.16** | **24.02** | **25.18** | **24.64** | **27.98** | **26.08** | **28.58** | **27.1** | **30.78** | **30.74** |
| **SD** | **0.5** | **0.7** | **1.4** | **0.8** | **1.8** | **2.8** | **1.6** | **0.8** | **0.7** | **1.8** | **1.9** | **0.7** | **1.9** | **1.9** | **0.8** | **1.4** |

**Table S1a related to Figure 1:** P-values of Tukey's pairwise comparison of biweekly weight of male mice to the weight on day 1 of the experiment p ˂ 0.05.

|  | **Day 1** | | **Week 2** | | **Week 4** | | **Week 6** | | **Week 8** | | **Week 10** | | **Week 12** | | **Week 14** | |
| --- | --- | --- | --- | --- | --- | --- | --- | --- | --- | --- | --- | --- | --- | --- | --- | --- |
|  | **CBD** | **AIN-93M** | **CBD** | **AIN-93M** | **CBD** | **AIN-93M** | **CBD** | **AIN-93M** | **CBD 8** | **AIN-93M 8** | **CBD 10** | **AIN-93M 10** | **CBD 12** | **AIN-93M 12** | **CBD 14** | **AIN-93M 14** |
| CBD Day 1 |  | 1 | 1 | 1 | 0.9939 | 0.9215 | 0.05072 | 0.5882 | 0.04787 | 0.1946 | 1.32E-06 | 0.002496 | 1.03E-07 | 4.98E-05 | 1.82E-11 | 1.98E-11 |
| AIN-93M Day 1 | 0.7492 |  | 1 | 0.997 | 1 | 0.997 | 0.1859 | 0.8988 | 0.1776 | 0.4987 | 1.06E-05 | 0.01392 | 8.62E-07 | 0.000358 | 8.09E-11 | 9.42E-11 |

**Key:**

CBD Cereal Based diet

AIN-93M American Institute of Nutrition-93 M diet

**Table S2 related to Figure 1:** Mean biweekly weights (g) of Female mice fed on either CBD or AIN-93M for 15 weeks

|  | **Day 1** | | **Week 2** | | **Week 4** | | **Week 6** | | **Week 8** | | **Week 10** | | **Week 12** | | **Week 14** | |
| --- | --- | --- | --- | --- | --- | --- | --- | --- | --- | --- | --- | --- | --- | --- | --- | --- |
|  | **CBD** | **AIN-93M 93M** | **CBD** | **AIN-93M 93M** | **CBD** | **AIN-93M 93M** | **CBD** | **AIN-93M 93M** | **CBD** | **AIN-93M 93M** | **CBD** | **AIN-93M 93M** | **CBD** | **AIN-93M 93M** | **CBD** | **AIN-93M 93M** |
| **Average** | **21.28** | **21.48** | **20.3** | **21.62** | **22.58** | **24.2** | **22.28** | **23.14** | **23.56** | **24.06** | **24.7** | **24.94** | **25.64** | **25.96** | **25.94** | **25.96** |
| **SD** | **0.4** | **0.5** | **1.6** | **2.3** | **1.3** | **1.5** | **2.3** | **2.1** | **1.5** | **1.8** | **1.6** | **2.3** | **1.5** | **1.4** | **1.4** | **1.4** |

**Table S2a related to Figure 1:** P-values of Tukey's pairwise comparison of biweekly weight of female mice to the weight on day 1 of the experiment p ˂ 0.05.

|  | **Day 1** | | **Week 2** | | **Week 4** | | **Week 6** | | **Week 8** | | **Week 10** | | **Week 12** | | **Week 14** | |
| --- | --- | --- | --- | --- | --- | --- | --- | --- | --- | --- | --- | --- | --- | --- | --- | --- |
|  | **CBD** | **AIN-93M** | **CBD** | **AIN-93M** | **CBD** | **AIN-93M** | **CBD** | **AIN-93M** | **CBD** | **AIN-93M** | **CBD** | **AIN-93M** | **CBD** | **AIN-93M** | **CBD** | **AIN-93M** |
| CBD Day 1 |  | 1 | 0.9999 | 1 | 0.9963 | 0.2877 | 0.9998 | 0.9099 | 0.6953 | 0.3661 | 0.1006 | 0.0554 | 0.007496 | 0.002711 | 0.002893 | 0.002711 |
| AIN-93M Day 1 | 0.2726 |  | 0.9987 | 1 | 0.9994 | 0.4028 | 1 | 0.9626 | 0.8135 | 0.4937 | 0.1583 | 0.09139 | 0.01374 | 0.005152 | 0.005488 | 0.005152 |

**Key:**

CBD Cereal Based diet

AIN-93M American Institute of Nutrition-93 M diet
